# Anti-Rabies Vaccine Compliance and Knowledge of Community Health Worker Regarding Animal Bite Management in Rural Area of Eastern India

**DOI:** 10.7759/cureus.14229

**Published:** 2021-03-31

**Authors:** Dinesh P Sahu, Preeti PS, Vikas Bhatia, Arvind K Singh

**Affiliations:** 1 Community Medicine and Family Medicine, All India Institute of Medical Sciences, Bhubaneswar, Bhubaneswar, IND; 2 Community Medicine and Family Medicine, All India Institute of Medical Sciences, Bibinagar, Bibinagar, IND

**Keywords:** compliance, health worker, animal bite management, rural india, anti-rabies vaccine, rabies immunoglobulin

## Abstract

Background

Rabies is a public health problem in developing countries like India accounting for the second-highest number of rabies-related deaths worldwide. Anti-rabies vaccine (ARV) is the only proven and effective way of preventing death in this 100% fatal disease. However, compliance is a real concern. This study aims to assess the compliance of ARV and rabies immunoglobulin (RIG) among the ARV-clinic beneficiaries and also the knowledge of the health workers regarding animal bite management.

Methods

A cross-sectional study was conducted in an ARV clinic of a community health centre in a rural Odisha (Eastern India) between February and April 2019. All the beneficiaries attending the ARV clinic were followed up for 28 days to assess their ARV and RIG compliance. Data were collected using a pre-designed semi-structured questionnaire and analysis was performed using SPSS v.22 (IBM Corp, Armonk, NY, USA). Proportion was calculated for categorical variables and mean for continuous variables. Chi-square test was applied to test for significance of categorical variables.

Results

A total of 468 beneficiaries were followed up. More than half (59.8%) of the animal bite victims had a category-II bite, followed by 33.4% having category-III, and 6.8% having a category-I bite. Around three-fourth were exposed to dog bite. Only 52.3% of the patients were compliant with ARV, and 49.4% were compliant with RIG. Knowledge of the health workers regarding wound management was found to be sub-optimal.

Conclusion

Poor ARV compliance was seen among the beneficiaries. Awareness activities need to be strengthened further to improve health-seeking behaviour. The significant gap in knowledge of the health workers indicates the need for professional training at regular intervals.

## Introduction

Rabies is a zoonotic disease with 100% fatality and is a public health burden. It is estimated that more than 1.4 billion people are at risk of rabies infection in the South-East Asia region. Each year, 23,000-25,000 people die due to the extremely fatal disease in this region, which accounts for 45% of the rabies death worldwide [1]. Around 36% of the world’s rabies death occur in India each year [2]. But this can be an underestimation as true rabies is not a notifiable disease in India [3]. Rabies fatalities in India are the highest in Asia and the second highest in the world [4]. It is endemic throughout the country with the exception of Andaman & Nicobar and Lakshadweep Islands [5]. India spends approximately INR 15 billion on rabies vaccines alone, exerting a sizeable economic burden on the government [6]. A major challenge in estimating the burden of rabies is the absence of reliable surveillance data for countries where the disease is most prevalent [3].

Even though deaths caused by rabies are preventable by the timely application of appropriate prophylaxis, mortality remains considerably high. This situation is rooted in the lack of awareness regarding preventive measures of rabies, proper post-exposure prophylaxis, and also poor access to proper health services. There are several myths in the community concerning initial wound management of animal bites, resulting in a delay in seeking post-exposure prophylaxis. Irregular supply of anti-rabies vaccine and immunoglobulin, particularly in primary healthcare facilities of rural India, is also responsible for the same [7]. Our awareness regarding the disease and its preventive measures can play a vital role in decreasing rabies-related morbidity and mortality. To address the rising burden of rabies in India, National Rabies Control Program (NRCP) emphasized rabies surveillance and ensuring the availability of ARV and immunoglobulin.

Post-exposure prophylaxis (PEP) with ARV for rabies is believed to be one of the cost-effective methods of preventing death due to dog bite. Lack of PEP and poor compliance to ARV are the important reasons for rabies death [8-10]. Only 47.9% of the dog bite victims received ARV and half of them received rabies immunoglobulin [11].

With the above-cited picture, a study was conducted to determine the demographic characteristics of patients attending ARV clinic, their wound management practices, and their adherence to ARV and RIG. Health workers’ knowledge regarding animal bite management was also assessed as they are the first point of contact for the rural community.

## Materials and methods

This cross-sectional study was conducted in an ARV clinic of Community Health Centre (CHC) Tangi, which is a rural health training center of All India Institute of Medical Sciences, Bhubaneswar, for the period of February to April 2019. A sample size of 423 was calculated assuming 50% ARV compliance, absolute precision of 5%, and a dropout rate of 10%. However, the universal sampling method was adopted to get 468 patients in the three months of the study period. This CHC receives on average 4-5 new animal bite cases and a total of around 20 animal bite cases for anti-rabies vaccination every day.

All the beneficiaries coming to the ARV clinic during this period were included in this study during their first visit and were prospectively followed up for the next 28 days for assessment of completion of the four doses of ARV and rabies immunoglobulin. All the ARV beneficiaries were interviewed for wound-washing practices during any of the visits; however, most of the beneficiaries were assessed in the first visit. All other data like demographic variables, the grade of bite, site of the bite, nature of bite (pet/stray), type of animal exposed, the status of ARV, immunoglobulin, and injection vaccination were assessed from the ARV register. As rabies immunoglobulin is not available at this health facility, the compliance to immunoglobulin was assessed only in the follow-up visits.

The beneficiaries who visited the health facility were first assessed by a physician to determine the category as well as the severity of the bite. After the initial evaluation, the beneficiaries were referred to the ARV clinic for wound management and vaccination. Animal bites are categorized into categories I, II, and III as per the type of contact. Category I is no exposure, or the animal licks while touching or feeding on intact skin; category II is nibbling of uncovered skin, or minor scratches or abrasion without bleeding; and category III is single or multiple transdermal bites or scratches, contamination of mucous membrane or broken skin with saliva from animal licks, or exposure due to direct contact with bats. ARV is administered for category II and III, and rabies immunoglobulin is administered for category III bite [12]. An intradermal schedule is followed in all public health facilities across the state.

Knowledge assessment regarding animal bite management was done for the Accredited Social Health Activists (ASHA) of the block (administrative area). The sample size was calculated assuming that 50% of the ASHAs have adequate knowledge of dog bite management with a relative precision of 20%. The finite correction was done as the total number of ASHAs was 162. The final sample size was calculated to be 60. ASHAs attending the monthly meetings were included in the study.

Data was collected using a pre-designed semi-structured questionnaire. The face and content validity of the questionnaire was established by revising the questionnaires following feedback from an expert. The respondents were not acquainted with the terms “anti-rabies vaccine” (ARV) and “anti-rabies serum;” (ARS); hence, they were asked about the site and the number of injections given. If the injection was administered at the site of the wound, we recorded it as ARS, while the data for ARV was collected from the records. For the knowledge assessment of the ASHAs, the questions were scored. For full and partial correct responses, the scores were 1 and 0.5, respectively. For wrong responses, a 0 score was given. 

A participant was defined as compliant for ARV when the participant had taken four doses of ARV and the participant was defined as compliant for RIG when the participant had taken RIG before the third dose (day 7th) of ARV. RIG compliance was verified from the prescription of the participants. For the re-exposure cases, the appropriate dosing schedule was considered instead of the full four doses.

The study was initiated following the clearance from the Institute Ethics Committee, All India Institute of Medical Sciences, Bhubaneswar. Data was collected and compiled in Microsoft Excel 2015 (Microsoft Corporation, Redmond, WA, USA) and then analyzed using Statistical Package for Social Sciences version 22 (IBM Corp, Armonk, NY, USA). All the categorical variables were expressed as percentage or proportion and the level of significance between two or more categorical variables was assessed by the Chi-square test. A p-value of less than 0.05 was considered as significant. Continuous variables are presented as mean and standard deviation.

## Results

A total of 468 animal bite beneficiaries turned out to the ARV clinic during the study period of three months. The mean age of the ARV beneficiaries was 31.52 ± 20.28, however, the beneficiaries were from the minimum age of 1 year to a maximum of 85 years. Around one-fourth (26.3%) of the beneficiaries were aged 0-14 years and one-third (33.3%) were above 40 years. Males were 67.7% (317) of the beneficiaries (Table 1).

**Table 1:** Demographic variables, exposure categorization and primary management of animal bite cases (N=468) ARV: anti-rabies vaccine, CHC: community health center *Others: face and trunk

		Compliant to ARV (N=228) n (%)	Not Compliant to ARV (N=240) n (%)	p-value
Gender	Male	148 (64.9)	169 (70.4)	0.235
	Female	80 (35.1)	71 (29.6)	
Age group	0-14 years	62 (27.2)	61 (25.4)	0.132
	15-40 years	100 (43.9)	89 (37.1)	
	>40 years	66 (28.9)	90 (37.5)	
Animal exposed	Dog	166 (73.1)	184 (76.7)	0.733
	Cat	39 (17.2)	34 (14.2)	
	Monkey	14 (6.2)	12 (5.0)	
	Others	8 (3.5)	10 (4.2)	
Type of animal exposed	Stray	206 (93.5)	215 (93.2)	0.910
	Pet	15 (6.5)	15 (6.8)	
Nature of bite	Intact skin	0 (0.0)	32 (13.3)	0.359
	Broken skin	151 (66.2)	129 (53.8)	
	Bleed	77 (33.5)	79 (32.9)	
Site of Bite	Lower limb	85 (37.2)	95 (39.5)	0.102
	Upper limb	52 (22.8)	58 (24.2)	
	Others*	2 (0.01)	2 (0.01)	
	Information not available	89 (39.0)	85 (35.4)	
Wound washing practice before ARV	With soap and water	15 (7.0)	18 (8.5)	0.860
	Not washed	193 (90.6)	191 (89.7)	
	Other applications	4 (1.9)	5 (2.3)	
Distance from CHC	< 10 km	125 (54.8)	137 (57.1)	0.623
	≥10 km	103 (45.2)	103 (42.9)	

Most of the ARV beneficiaries (74.8%) were exposed to dog bite, followed by cat (15.6%) and monkey (5.6%). Only 3.8% of the participants had exposure to other animals such as bears, wolves, and bats. Most of the animals (90%) were stray in nature. The nature of bites is presented in Table 1. On the basis of WHO wound classification, more than half (59.8%) of the bites were of category-II, 6.8% of the bites were falling under category-I, whereas 33.4% were of category-III (Table 1).

Before attending the ARV clinic, only 7.1% of the animal bite victims reported having washed their wounds with running tap water or with soap. The majority of the victims (82.1%) did not wash the wound at all and few victims reported an inappropriate application of coconut water, plant extracts, and turmeric (Table 1).

Around half (52.3%) of the animal bite patients attending the ARV clinic completed their schedule of four doses of ARV. Similarly, 25.5% of the beneficiaries completed three doses, 14.4% completed two doses, and 7.8% took only one dose of ARV. 81.2% of the animal bite patients received tetanus toxoid injection. Out of the 156 beneficiaries who required RIG, only half (77, 49.4%) had taken it (Table 2). Most of the patients who did not take RIG were from the age group of more than 40 years. We could not find any significant difference in ARV compliance with age, category of bite, site of the bite, type of animal exposed, and distance from the health center (Table 1). Similarly, for compliance of RIG, gender was found to have a significant association, and all other factors like age, type of animal exposed, category of bite, distance from the hospital, wound washing practices, and ARV compliance were found to have no association with RIG compliance (Table 3).

**Table 2:** ARV compliance among animal bite victims (N=436) ARV: anti-rabies vaccine, RIG: rabies immunoglobulin, TT: tetanus toxoid

ARV compliance (N=436)	Frequency	Percent
One dose	34	7.8
Two doses	63	14.4
Three doses	111	25.5
Full doses	228	52.3
TT compliance (n=436)	Frequency	Percent
Not taken	54	12.5
Taken	378	87.5
RIG compliance (n=156)	Frequency	Percent
Not taken	79	50.6
Taken	77	49.4

**Table 3:** RIG compliance and its associations (N=156) ARV: anti-rabies vaccine, RIG: rabies immunoglobulin *Others: bear bite

		RIG not taken (N=79) n (%)	RIG taken (N=77) n (%)	p-value
Age group	0-14 yrs	15 (19.0)	21 (27.3)	0.201
	15-40 yrs	19 (24.1)	23 (29.9)	
	>40 yrs	45 (56.9)	33 (42.8)	
Gender	Male	50 (63.3)	60 (77.9)	0.045
	Female	29 (36.7)	17 (22.1)	
Type of animal exposed	Stray	74 (93.7)	68 (88.3)	0.486
	Pet	5 (6.3)	9 (11.7)	
Animal exposed	Dog	62 (78.5)	64 (83.1)	--
	Cat	13 (16.4)	8 (10.4)	
	Monkey	4 (5.1)	4 (5.2)	
	Others*	0 (0.0)	1 (1.3)	
Category of bite	Category 1	0 (0.0)	0 (0.0)	----
	Category 2	0 (0.0)	0 (0.0)	
	Category 3	79 (100.0)	77 (100.0)	
Distance	Distance <10 km	41(51.9)	51(66.2)	0.068
	Distance >10 km	38(48.1)	26(37.8)	
Wound washing practices	With soap and water	4 (5.0)	5 (6.5)	0.785
	Not washed	66 (83.5)	61 (79.2)	
	Other applications	9 (11.4)	11 (14.3)	
ARV compliance	ARV compliant	38 (48.1)	39 (50.6)	0.752
	ARV non-compliant	41 (51.9)	38 (49.4)	
	Total	79(100.0)	77(100.0)	

A total of 64 health workers were interviewed to assess the knowledge regarding rabies and its primary management. The majority of the health workers 98.4% (63 out of 64) had heard of rabies as a disease and 81.3% (52 out of 64) considered rabies as a fatal disease. Around two-thirds of the health workers felt that rabies was a serious problem in their area. Knowledge regarding the mode of transmission of the disease was assessed. Animal bite was found to be the most common (85.9%) mode of transmission as answered by the respondents. Other than dog, pig (62.5%), cat (42.2%), rat (15%), monkey (12.5%), fox (10.9%), and bear (10.9%) were the animals transmitting rabies. Head was considered as the most dangerous site of the bite by 62.5% of the health workers. Knowledge regarding wound management of animal bites was found to be sub-optimal among the health workers. Two-thirds of the health workers mentioned tying with a rope as one of the commonest methods of wound management. Only 23.4% of the health workers correctly stated washing with soap and water after animal bite as primary wound management. Tetanus toxoid and anti-rabies vaccine were considered essential for preventing rabies death after an animal bite by 92.2% of the health workers. Only half of the health workers could correctly mention the nearest hospital where ARV was available (Table 4). 

**Table 4:** Health worker knowledge regarding rabies and primary management (N=64) * Multiple responses possible

		n (%)
Heard of rabies	Yes	63 (98.4)
	No	1 (1.6)
Is rabies a fatal disease	Yes	52 (81.3)
	No	8 (12.5)
	Don’t know	4 (6.3)
Rabies as a serious problem of their area	Yes	42 (65.6)
	No	17 (26.6)
	Don’t know	5 (7.8)
Mode of transmission*	Bite	55 (85.9)
	Saliva	44 (68.8)
	Scratching	22 (34.4)
	Eating raw meat	2 (3.1)
Animal other than dog transmitting the disease*	Pig	40 (62.5)
	Cat	27 (42.2)
	Rat	15 (23.4)
	Monkey	8 (12.5)
	Fox	7 (10.9)
	Bear	7 (10.9)
Danger site of bite*	Head	40 (62.5)
	Face	26 (40.6)
	Genitalia	9 (14.1)
	Neck	8 (12.5)
	Limbs	3 (4.7)
	Trunk	3 (4.7)
First aid wound management for animal bite*	Tie with rope	43 (67.2)
	Wash with soap	15 (23.4)
	Wash without soap	13 (20.3)
	Apply turmeric/oil/sandalwood	10 (15.6)
	Apply antiseptic	2 (3.1)
Necessity of TT injection	Yes	59 (92.2)
	Don’t know	5 (7.8)
Necessity of ARV after bite	Yes	59 (92.2)
	Don’t know	5 (7.8)
Site of availability of ARV*	Sub centre	0
	Primary health centre	31 (48.4)
	Community health centre	33 (51.6)
	District hospital	35 (54.7)
Knowledge score of health workers	Below 6.5	30 (46.8)
	6.5 or more	34 (53.2)

## Discussion

In the current study, males were the predominant victims contributing to 66.7% of the cases while 33.3% were females. A similar result was reported by Dhaduk et al. in a study done in Gujarat, where the male and female ratio was 3:1 [13]. This male predominance could probably be because males are more involved in outdoor activities in comparison to females. A multi-centric study by Sudarshan et al. in India revealed similar findings [14]. 26.3% were children and 40.4% were from 15-40 years. Similarly, children of <15 years and youth of 15-30 years were the predominant participants in the study done by Dhaduk et al. [12]. Children indulged more in playful activities and this makes them more vulnerable to animal bites, and their lower ability to react to any kind of threat situation makes them susceptible to falling prey to the animals [9]. Similar findings were observed by Domple et al. in a hospital-based study done in Maharashtra, Karthik et al. in Bangalore, and Sudarshan et al. in a multi-centric study in India [15-17].

The most common animal bite reported in our study was by dogs, followed by cats and monkeys; and the majority of the animals (90%) were stray. A similar finding was reported in a multi-centric study in India [14,17,18]. The similarity in results can be explained by the similar study population, also all were hospital-based study. This result corroborates the national data on animal bite causing rabies that stated that 96% of the rabies is due to dog bites [3]. Therefore, dog population management by animal birth control can be adopted.

As the study was done at the ARV clinic of a community health center, very few (6.8%) of the patients reporting were of category-I bite. In the current study, the majority of the participants were of category-II, then category-III. A similar result was reported by Salahuddin et al. in Pakistan where 11.9% were category-III and 42.7% were category-II bites [19]. This result was contrasting with other studies done in this respect [10,18,20]. But the result was similar to that of a community-based study by Agarvval et al. and Sahu et al. in Lucknow [21,22]. The lower limb was the commonest site of animal bite followed by the upper limb. Similar results were seen in various studies done across the country [14,15,18,22,23].

Most of the victims in our study reported not having washed their wounds after an animal bite. This shows that most of them were not aware of the primary wound management procedure. This result was similar to that obtained by Anandaraj et al. [18]. This displays a dire need for a generation of public awareness about rabies and primary wound management following an animal bite.

Only half of the victims were compliant with ARV in our study. Similar results were observed in other Indian studies [10,16,20,22]. A higher percentage of ARV compliance was observed by Anandaraj et al. in Karnataka and Domple et al. in Maharastra [15,18]. Compliance of ARV in the intradermal route (77%) was found to be significantly higher than the intramuscular route (60%) in a study done by Shankaraiah et al. at Bangalore [24]. Our study did not reveal any significant difference between those who were compliant and those who were not. Though the intradermal schedule is followed in public health facilities of Odisha, compliance in the current study was lower than that of Shankaraiah et al. This implies the knowledge gap regarding rabies and the need for ARV among the beneficiaries. Health education and sensitization in the above context are vital for control and prevention of the fatal disease as noncompliance in the ARV schedule can be attributed to the lack of awareness of the need to complete the full doses.

Only half of the beneficiaries who required RIG were compliant and most of them were from the younger and middle age group, poor compliance was predominantly observed among those of extreme ages. This can be elucidated based on the fact that they were dependent on others to take them to a health facility with the availability of RIG. As RIG is essential in the case of category-III bites, it should be made available at all community health facility levels, or wherever the caseload of dog bites is high.

Community health workers can act as an important link in the control of the deadly disease as they are part of the same community and are the first line of contact to the health system. Almost all the community health workers were aware of rabies and its fatality. However, there exist some knowledge gaps amongst the health workers. For instance, regarding the mode of spread of disease, where only 85.9% responded bite, 68.8% for saliva, 34.4% for scratching as the mode of transmission. This indicates inadequate knowledge among the community health workers. And a pressing need for refresher training for the community health workers at frequent intervals. Also, they were not aware of any other animals transmitting rabies, other than the dog. A study amongst the health workers in North Vietnam named dogs as the most common source of rabies but unlike participants in our study, most participants (94%) correctly responded that cats, bats, and ferret-badgers were other sources of rabies [19]. Proper knowledge about animals causing rabies can be crucial in avoiding rabies-like dreadful consequences. Although it was good to note that our study participants knew head, face, and genitalia as danger sites of an animal bite. This implies that they have some knowledge regarding rabies but knowledge about first-aid management after the animal bite was appalling. Most of them considered tying a rope proximal to the wound, which is inappropriate management. They also suggested practices of applying various substances like turmeric, oil, or sandalwood to the wound. This confirms that wrong practices still prevail in the community owing to a lack of awareness about initial wound management. Contrasting with our findings, the all the health workers in the study by Tiwari et al. stated that traditional drugs should not be given [25].

A study reported that none of the respondents would have given traditional drugs and nearly one-quarter would have covered the wound with traditional drugs/herbs. The reason for reliance on this unproven method of treatment was not identified in this study. Even though public health workers are aware that vaccine is required, embedded local beliefs, practices, and culture persist, which contributes to wrong, unhygienic practices of wound management and delay in seeking medical attention. Other potential factors may be poor access to health services. Tetanus toxoid injection and anti-rabies vaccine were considered necessary by the health workers. Half of the community health workers were unaware of the availability of the anti-rabies vaccine in the community health center. As community health workers are the first point of contact for the community, they must be aware of the availability of anti-rabies vaccine in the nearest health facility. Therefore, community health workers should be explained about the disease, mode of spread, severity, and management to prevent rabies-related mortality.

The study is the first of its kind in Eastern India among the rural population. The study was conducted in a sufficient sample size. The major limitation of the study is that it was a hospital-based study. The animal bite victims not reporting to hospitals were missed. As this is the only hospital in the area having the ARV facility, the results can be generalized.

## Conclusions

Our study revealed that compliance to ARV was poor among the beneficiaries, where only half of them remain compliant to all four doses. This is a serious concern as the vaccination is available free of cost. Poor compliance can be attributed to a lack of knowledge about the importance of completing the schedule. Poor compliance to RIG could be attributed to non-availability. RIG compliance was poor among the extreme age groups, which can be due to the dependency of the younger and older people to reach the higher center for availing RIG. Another concerning finding was the significant gap in the knowledge about prevention and control of rabies among health workers. Public health workers have a crucial role to play in the prevention of rabies as they can generate awareness in the community about the right practices for animal bite management. Given the insights obtained from this study, awareness activities need to be strengthened further to improve health-seeking behavior. The policies should be directed towards increasing availability and accessibility to RIG. The need for the continued professional training of public health workers at regular intervals has to be addressed.
